# The SMART Registry: Long-Term Results on the Utility of the Penumbra SMART COIL System for Treatment of Intracranial Aneurysms and Other Malformations

**DOI:** 10.3389/fneur.2021.637551

**Published:** 2021-04-13

**Authors:** Alejandro M. Spiotta, Min S. Park, Richard J. Bellon, Bradley N. Bohnstedt, Albert J. Yoo, Clemens M. Schirmer, Reade A. DeLeacy, David J. Fiorella, B. Keith Woodward, Harris E. Hawk, Ashish Nanda, Osama O. Zaidat, Peter J. Sunenshine, Kenneth C. Liu, Mouhammed R. Kabbani, Kenneth V. Snyder, Thinesh Sivapatham, Travis M. Dumont, Alan R. Reeves, Robert M. Starke, The SMART Registry Investigators

**Affiliations:** ^1^Department of Neurosurgery, Medical University of South Carolina, Charleston, SC, United States; ^2^Department of Neurosurgery, University of Virginia, Charlottesville, VA, United States; ^3^Department of Interventional Neuro Radiology, Swedish Medical Center, Denver, CO, United States; ^4^Oklahoma University Medical Center, Oklahoma City, OK, United States; ^5^Department of Interventional Neuroradiology, Texas Stroke Institute, Dallas, TX, United States; ^6^Geisinger Medical Center, Danville, PA, United States; ^7^Mount Sinai, New York, NY, United States; ^8^Stony Brook University Medical Center, Cerebrovascular Center, New York, NY, United States; ^9^Vista Radiology, Knoxville, TN, United States; ^10^Erlanger Health System, Chattanooga, TN, United States; ^11^SSM Health Medical Group, Fenton, MO, United States; ^12^St Vincent Mercy Health Medical Center, Toledo, OH, United States; ^13^Banner University Medical Center, Phoenix, AZ, United States; ^14^Penn State Milton S. Hershey Medical Center, Hershey, PA, United States; ^15^Gundersen Health System, La Crosse, WI, United States; ^16^Department of Neurosurgery, University of Buffalo, Buffalo, NY, United States; ^17^Christiana Care Health System, Newark, DE, United States; ^18^Department of Surgery, University of Arizona, Tucson, AZ, United States; ^19^Department of Radiology, University of Kansas, Kansas City, KS, United States; ^20^Department of Neurological Surgery, University of Miami Hospital, Miami, FL, United States

**Keywords:** embolization coil, intracranial aneurysm, SMART COIL, intracranial fistula, intracranial malformations

## Abstract

**Introduction:** Penumbra SMART COIL® (SMART) System is a novel generation embolic coil with varying stiffness. The study purpose was to report real-world usage of the SMART System in patients with intracranial aneurysms (ICA) and non-aneurysm vascular lesions.

**Materials and Methods:** The SMART Registry is a post-market, prospective, multicenter registry requiring ≥75% Penumbra Coils, including SMART, PC400, and/or POD coils. The primary efficacy endpoint was retreatment rate at 1-year and the primary safety endpoint was the procedural device-related serious adverse event rate.

**Results:** Between June 2016 and August 2018, 995 patients (mean age 59.6 years, 72.1% female) were enrolled at 68 sites in the U.S. and Canada. Target lesions were intracranial aneurysms in 91.0% of patients; 63.5% were wide-neck and 31.8% were ruptured. Adjunctive devices were used in 55.2% of patients. Mean packing density was 32.3%. Procedural device-related serious adverse events occurred in 2.6% of patients. The rate of immediate post-procedure adequate occlusion was 97.1% in aneurysms and the rate of complete occlusion was 85.2% in non-aneurysms. At 1-year, the retreatment rate was 6.8%, Raymond Roy Occlusion Classification (RROC) I or II was 90.0% for aneurysms, and Modified Rankin Scale (mRS) 0-2 was achieved in 83.1% of all patients. Predictors of 1-year for RROC III or retreatment (incomplete occlusion) were rupture status (*P* < 0.0001), balloon-assisted coiling (*P* = 0.0354), aneurysm size (*P* = 0.0071), and RROC III immediate post-procedure (*P* = 0.0086) in a model that also included bifurcation aneurysm (*P* = 0.7788). Predictors of aneurysm retreatment at 1-year was rupture status (*P* < 0.0001).

**Conclusions:** Lesions treated with SMART System coils achieved low long-term retreatment rates.

**Clinical Trial Registration:** https://www.clinicaltrials.gov/, identifier NCT02729740.

## Introduction

Intracranial aneurysm rupture is a catastrophic clinical outcome and occurs annually in ~30,000 people in the U.S. (1) Endovascular coiling is widely accepted as treatment for intracranial aneurysms following the results from ISAT (2). Endovascular coiling was shown to be safe and efficacious and resulted in less disability than surgical clipping. However, retreatment rate at 1-year was higher for coiling (17.4%) than for surgical clipping (3.8%) (3). In the decade since the ISAT results were published, advances in coiling technology, including deliverability, materials, and design, have been developed. Endovascular coiling remains a viable treatment alternative to flow diverters and intrasaccular devices.

The Penumbra SMART COIL® (SMART) System is a microcoil system, which includes SMART COIL®, Penumbra Coil 400™ (PC400™), and Penumbra Occlusive Device® (POD®) indicated for endovascular embolization in the peripheral and neuro vasculature. All devices were available in the U.S market at the start of enrollment in 2016. The SMART System's unique design feature is varying levels of softness that increase toward the proximal tip. This progressive softness enhances deliverability in multiple aneurysm sizes and shapes. The SMART System design may improve deployment by stabilizing the microcatheter positioning, compared to other coils with uniform stiffness (4). The unique design of the SMART System may possibly assist with achieving higher packing density and adequate occlusion in multiple aneurysm types.

Other SMART Coil study results reported adequate aneurysm occlusion between 71.2 and 100% of treated aneurysms, low rebleeds, and low retreatment rates (5–8). Those studies had either small sample sizes, few study sites, or lacked long-term follow-up. The SMART Registry was a prospective, post-market study, designed to evaluate the SMART System in the treatment of intracranial aneurysms and other neurovascular lesions. We report the results of the SMART Registry procedural and long-term study endpoints in patients with intracranial aneurysm or other, non-aneurysm lesions, and investigate predictors of aneurysm retreatment and inadequate occlusion at 1-year. The SMART Registry provides valuable insights on treatment practices in the U.S. and real-world data on common safety and efficacy outcome measures.

## Materials and Methods

### Design

The SMART Registry was a prospective, single-arm, post-market, multicenter registry of the SMART System (Penumbra, Inc.). A maximum of 1,000 patients were planned to be enrolled in up to 100 international sites. Patients were treated in accordance with the cleared indications for the SMART, PC 400, and POD for intracranial aneurysms and other, non-aneurysm vascular lesions. The study received approval from the Institutional Review Board (IRB) and Ethics Committee (EC) from each participating site with oversite for the duration of the registry. The study was designed in accordance with the relevant aspects of clinical research regulations. Written informed consent was provided by patients or their legally authorized representative (LAR). For patients that were treated in emergent cases, they were allowed to be enrolled if they signed the consent within one calendar day after the procedure, or if a LAR signed on their behalf. Penumbra, Inc. sponsored the oversite of this trial.

### Eligibility Criteria

Patients included in the SMART Registry were required to have a signed consent form and undergo embolization of an intracranial aneurysm or other neurovascular abnormalities such as an arteriovenous malformations or an arteriovenous fistula (non-aneurysm lesions) according to the cleared indications. Patients were excluded if SMART, PC400, or POD coils accounted for <75% of the total number of coils implanted; if their life expectancy was <1 year; if they were already enrolled in the SMART Registry, or if they were participating in another investigation(s) that could confound results.

#### Endpoints

The primary efficacy endpoint was retreatment at 1-year (±6 months), and the primary safety outcome was procedural device-related serious adverse events (SAEs) within 24 h of the procedural arterial puncture. The secondary efficacy endpoints were ability to achieve adequate occlusion immediate post-procedure and the number of times re-access with the microwire was required due to catheter kickout. Other clinical outcomes were adequate occlusion, recanalization, and modified Rankin Scale (mRS) 0-2 at 1-year; all-cause mortality within 24 h of procedure and at 1-year; and all SAEs that were intraprocedural or within 24 h of procedure, after 24 h and up to 365 days (±180 days for visits in window).

### Procedures and Data Collection

Demographics and medical history were recorded at the baseline visit and all patients were assessed in accordance with the institutional standard of care. Endovascular embolization procedures were completed per each investigational site's standard of care. Coil diameter and lengths were selected on the basis of aneurysm dimensions and physician preference. All lesions were treated with ≥75% SMART coils. Patients were administered local, conscious, or general sedation per physician preference. Target lesions were accessed by transfemoral, trans-radial, or other approaches. All adjunctive techniques and devices were permitted. Cerebral angiograms were obtained immediate post-procedure and at 1-year (±6 months). The aneurysm occlusion status was determined by the treating physician immediately post-procedure and the results were used to compare to the follow-up assessment. From the time of enrollment through study exit, safety endpoints were monitored, and adverse events were collected and assessed for whether they were serious, device related, or procedure related. Study data was collected by investigational sites using Inform electronic data capture (EDC) system.

### Study Definitions

The RROC was used to determine angiographic aneurysm occlusion status, with Class I as complete occlusion, Class II as residual neck, and Class III as residual aneurysm (9). For non-aneurysm lesions, occlusion was recorded as complete or incomplete as determined by the treating physician's assessment of whether blood flow remained on post-procedure imaging. The Hunt and Hess scale (10) was used at admission to determine severity of ruptured aneurysms; all scores were eligible for enrollment. For device-related and procedure-related SAEs, relationship to the device (definite/probable/possible/unrelated) was determined by the investigator. Events reported as “definite, probable, or possible” were classified as “related.” Coil packing density was calculated by using either software calculators or by calculating aneurysm volume assuming an ellipsoid model and coil volume [V = π (*p*/2)^2^ × L], where *p* represents primary coil diameter, and L represents coil length. Packing density was not calculated for patients with deconstructive treatment, fusiform or dissecting aneurysms, or non-aneurysmal lesions. A wide-necked aneurysm was defined as having a dome-to-neck ratio < 2 or a neck width of at least 4 mm. The Modified Rankin Scale (mRS) was captured at admission and at 1-year if available per the site standard of care.

### Statistical Analysis

The predetermined sample size (*n* = 1,000) allowed the expected retreatment rate of 8.3% at 1 year to be estimated with > ± 2% precision. Summary statistics for all patients, patients with aneurysm, and patients with non-aneurysm lesions were developed separately. Descriptive statistics with a 95% two-sided confidence interval were presented for most analyses. Continuous variables were summarized with descriptive statistics [*n*, mean, standard deviation, median, and interquartile range (IQR)]. For categorical data, frequency counts and percentage of patients within each category were included. Predictive analyses were performed for 1-year outcomes after aneurysm treatment—incomplete occlusion (RROC III or retreatment) and retreatment. The analyses were run by using binary logistic regression models with stepwise selection (alpha-to-enter ≤ 0.20, alpha-to-leave > 0.05). The following variables were considered for predictive models when their univariate logistic regression *p*-value was ≤ 0.20: patient age, sex, history of hypertension history of smoking, cocaine use, and family history of aneurysm or malformation; aneurysm type, size, and location; aneurysm status as ruptured, wide-necked, and neck width at least 4 mm; stent-assisted coiling; balloon-assisted coiling; deconstructive treatment; coil packing density; and RROC immediately post-procedure. SAS 9.4 (SAS Institute) was used for statistical programming.

## Results

### All Patient Outcomes

#### Demographics and Baseline Characteristics

**Figure 1:**
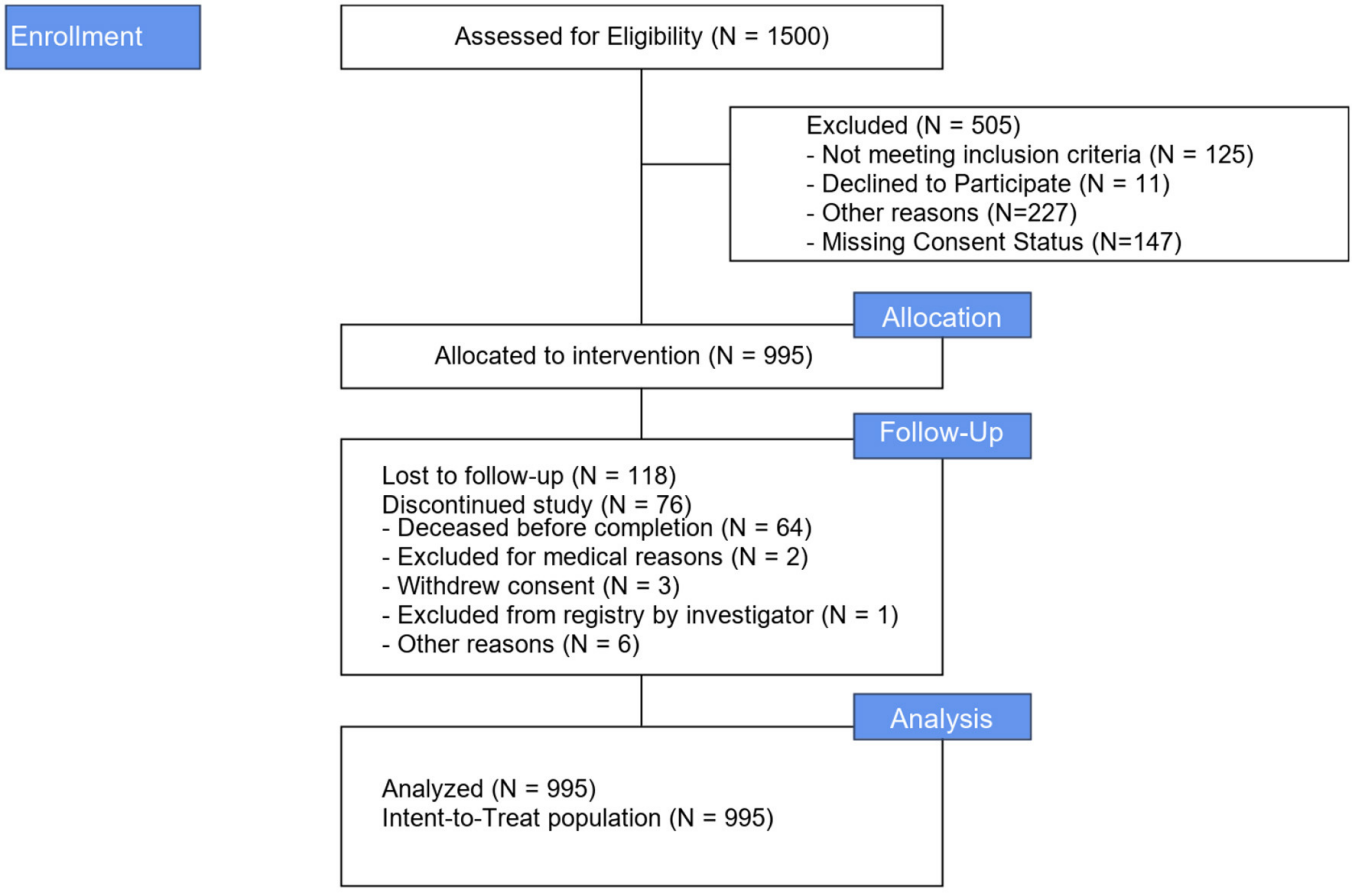
Study flow diagram.

Between June 2016 and August 2018, 995 patients were enrolled at 67 US sites (989 patients) and one Canadian site (6 patients). Figure 1 illustrates the study Flow Diagram. Baseline characteristics and medical history for all patients and by aneurysm and non-aneurysm cohort are presented in Table 1. The target lesion was an intracranial aneurysm in 905 (91.0%) patients; the remaining 90 (0.9%) lesion types were arteriovenous fistula, arteriovenous malformation, and vessel sacrifice or other pseudoaneurysm. For the overall population, mean age of patients was 59.6 years (SD 13.00, range 12–93 years), 72.1% of the patients were female, 61.2% had a history of smoking, 60.6% had a history of hypertension, and 17.1% had a family history of aneurysm or malformation.

**Table 1:** Demographics and baseline characteristics.

	**All patients** ** (*N* = 995)**	**Aneurysm patients** ** (*N* = 905)**	**Non-aneurysm patients** ** (*N* = 90)**
Age, years [average (SD)]	59.6 (13.00) (*N* = 995)	59.8 (12.64) (*N* = 905)	57.9 (16.18) (*N* = 90)
Age ≥ 65 years	38.3% (381/995)	38.0% (344/905)	41.1% (37/90)
Female	72.1% (717/995)	74.7% (676/905)	45.6% (41/90)
Hispanic or latino ethnicity	10.1% (33/326)	10.4% (31/299)	7.4% (2/27)
**Race**			
American Indian or Alaska Native	1.2% (4/326)	1.0% (3/299)	3.7% (1/27)
Asian	1.8% (6/326)	2.0% (6/299)	0.0%
Black or African American	13.2% (43/326)	12.7% (38/299)	18.5% (5/27)
White	78.5% (256/326)	78.6% (235/299)	77.8% (21/27)
Native Hawaiian or Other Pacific Islander	0.0%	0.0%	0.0%
Other	5.2% (17/326)	5.7% (17/299)	0.0%
**Medical history**			
Previous hemorrhagic stroke	8.8% (88/995)	9.6% (87/905)	1.1% (1/90)
Previous ischemic stroke	6.1% (61/995)	6.3% (57/905)	4.4% (4/90)
Hypertension	60.6% (603/995)	61.8% (559/905)	48.9% (44/90)
Family history of aneurysm or malformation	17.1% (170/995)	18.3% (166/905)	4.4% (4/90)
Diabetes	14.1% (140/995)	14.1% (128/905)	13.3% (12/90)
Smoking (current or former)	61.2% (609/995)	63.0% (570/905)	43.3% (39/90)
Polycystic kidney disease	0.6% (6/995)	0.7% (6/905)	0.0%

Lesion characteristics are described in Table 2. Most (75.2%) lesions were located in the anterior circulation. The lesion was on the left side in 35.6% patients, on the right side in 35.6% patients, and on the midline in 28.8% patients. Lesion locations included 3.7% (37/995) extradural ICA, 33.0% (328/995) ICA, 26.7% (52/995) ACA, 11.8% (117/995) MCA, 18.9% (188/995) posterior circulation, 2.4% (24/995) venous circulation, and 3.5% (35/995) other.

**Table 2:** Lesion characteristics.

	**All patients** ** (*N* = 995)**	**Aneurysm patients** ** (*N* = 905)**	**Non-aneurysm patients** ** (*N* = 90)**
Fistula	—	—	45.6% (41/90)
Arteriovenous malformation	—	—	12.2% (11/90)
Vessel sacrifice or pseudoaneurysm	—	—	42.2% (38/90)
**Lesion location**			
Extradural ICA	3.7% (37/995)	2.2% (20/905)	18.9% (17/90)
Intradural ICA	33.0% (328/995)	36.1% (327/905)	1.1% (1/90)
ACA	26.7% (266/995)	29.3% (265/905)	1.1% (1/90)
MCA	11.8% (117/995)	12.5% (113/905)	4.4% (4/90)
Posterior circulation	18.9% (188/995)	19.8% (179/905)	10.0% (9/90)
Venous circulation	2.4% (24/995)	0.1% (1/905)	25.6% (23/90)
Other	3.5% (35/995)	0.0%	38.9% (35/90)
**Lesion laterality**			
Left	35.6% (354/995)	33.9% (307/905)	52.2% (47/90)
Right	35.6% (354/995)	34.8% (315/905)	43.3% (39/90)
Midline	28.8% (287/995)	31.3% (283/905)	4.4% (4/90)
**Aneurysm characteristics**			
Aneurysm size, mm [average (SD)]	—	6.9 (3.62) (*N* = 905)	—
Aneurysm size group			
Small ( ≤ 4 mm)	—	19.0% (172/905)	—
Medium (>4–10 mm)		66.3% (600/905)	
Large (>10–25 mm)	—	14.5% (131/905)	—
Giant (>25 mm)	—	0.2% (2/905)	—
**Aneurysm type**			
Saccular	—	86.3% (778/901)	—
Dissecting		2.6% (23/901)	
Fusiform		2.4% (22/901)	
Other		8.7% (78/901)	
Wide neck	—	63.5% (554/872)	—
Ruptured	—	31.8% (288/905)	—
Hunt and Hess ≥ 3	—	43.5% (123/283)	—

#### Procedural Characteristics

Procedural characteristics are described in Table 3. The mean time from first coil deployment to last coil detached was 24.1 min (SD 25.73) and the median time was 16.0 (IQR 8.0, 32.0).

**Table 3:** Procedural characteristics.

	**All patients** ** (*N* = 995)**	**Aneurysm patients** ** (*N* = 905)**	**Non-aneurysm patients** ** (*N* = 90)**
**Adjunctive device use**			
Unassisted coiling	44.8% (446/995)	43.3% (392/905)	60.0% (54/90)
Stent-assisted coiling	34.4% (342/995)	37.2% (337/905)	5.6% (5/90)
Balloon-assisted coiling	18.7% (186/995)	20.3% (184/905)	2.2% (2/90)
Flow diverter	1.6% (16/995)	1.8% (16/905)	0.0% (0/90)
Liquid embolic	2.1% (21/995)	0.1% (1/905)	22.2% (20/90)
Particulate/plug embolic	1.2% (12/995)	0.3% (3/905)	10.0% (9/90)
Number of coils implanted [SMART/PC400/POD, average (SD)]	5.4 (5.03) (*N* = 994)	5.0 (3.88) (*N* = 904)	9.3 (10.62) (*N* = 90)
Packing density, % [average (SD)]	—	32.3 (18.21) (*N* = 819)	—
**Procedural times**			
Fluoroscopic time, min [median (IQR)]	37.0 (24.0, 56.0) (*N* = 992)	37.0 (24.0, 55.0) (*N* = 902)	41.0 (25.0, 73.0) (*N* = 90)
Time from first coil deployment to last coil detachment, min [median (IQR)]	16.0 (8.0, 32.0) (*N* = 952)	16.0 (8.0, 31.0) (*N* = 868)	20.5 (3.0, 53.0) (*N* = 84)

#### Performance Outcomes

Adequate occlusion at immediate post-procedure was achieved in 97.1% (965/994) of patients. The first framing coil was completely conformed to the lesion morphology in 83.1% (766/922) of cases. At 1-year, retreatment occurred in 6.8% (53/784) of patients and 83.1% (463/557) had mRS 0-2. Patient outcomes are provided in Table 4.

**Table 4:** Study outcomes.

	**All patients**	**Aneurysm patients**	**Non-aneurysm patients**
	**(*N* = 995)**	**(*N* = 905)**	**(*N* = 90)**
**Peri-procedural outcomes**			
Procedural device-related serious adverse events (SAEs)	2.6% (26/995)	2.9% (26/905)	0.0%
All SAEs (intraprocedural or within 24 h)	9.4% (94/995)	9.7% (88/905)	6.7% (6/90)
Mortality within 24 h of procedure	0.2%^*^ (2/995)	0.1% (1/905)	1.1% (1/90)
Adequate occlusion at immediate post-procedure		97.1% (879/905)	
Complete angiographic occlusion immediate post-procedure	—	—	85.2% (23/27)
Re-access attempts with guidewire due to catheter kickout (SMART/PC400/POD)			
0	81.7% (811/993)	80.7% (729/903)	91.1% (82/90)
1	10.6% (105/993)	11.1% (100/903)	5.6% (5/90)
2+	7.8% (77/993)	8.2% (74/903)	3.3% (3/90)
**Outcomes at 1-year**			
Retreatment	6.8% (53/784)	7.1% (52/731)	1.9% (1/53)
RROC I or II	—	90.0% (641/712)	—
Recanalization	12.4% (94/757)	12.9% (91/708)	6.1% (3/49)
mRS 0–2	83.1% (463/557)	84.4% (429/508)	69.4% (34/49)
All-cause mortality	6.0% (60/995)	5.4% (49/905)	12.2% (11/90)
All serious adverse events			
After 24 h through 365 days^†^	22.7% (226/995)	22.2% (201/905)	27.8% (25/90)

* *Not procedure-related or device-related*.

† *Defined as adverse events that started after the date of registry completion*.

#### Safety Outcomes

Procedural device-related SAEs occurred in 2.6% (26/995) of patients. The rate of device malfunctions associated with an AE was 0.2% (2/995); one patient experienced an aneurysm perforation and one patient experienced a coil herniation into the parent artery. The overall mortality rate for all patients within 24 h of the procedure was 0.2% (2/995). Neither of the two deaths were related to the device or the index procedure. The 1-year mortality rate was 6.0% (60/995). The SAE rate was 9.4% (intraprocedural or within 24 h) and 22.7% after 24 h through 365 days.

### Aneurysm Cohort Outcomes

#### Demographics and Baseline Characteristics

Of the 905 patients treated for aneurysms, the mean age was 59.8 year (SD 12.64), 74.7% were female, and 78.6% were white. There were 18.3% (166/905) of patients that had a familial history of aneurysms and 61.8% (559/905) had hypertension.

#### Procedural Characteristics

Average aneurysm size was 6.9 mm (SD 3.62) and 19.0% of aneurysms were small ( ≤ 4 mm). Patient aneurysm types were 86.3% saccular, 63.5% wide-necked, and 31.8% ruptured; 43.5% of patients with a ruptured aneurysm had Hunt and Hess ≥ 3 severity. Unassisted coiling (UAC) was used in 43.3% (392/905) of cases, stent-assisted coiling (SAC) was used in 37.2% (337/905) and balloon-assisted coiling (BAC) was used in 20.3% (184/905). The mean packing density was 32.3% (SD 18.21). The median time from first coil deployment to last coil detachment was 16.0 min (IQR 8.0, 31.0) and the median fluoroscopy time was 37.0 min (IQR 24.0, 55.0). In 80.7% (729/903) of cases, no re-access attempt was made due to catheter kick-out.

#### Performance Outcomes

Adequate occlusion at immediate post-procedure was achieved in 97.1% (879/905) of patients. Catheter kick-back (requiring re-access at least once or twice) occurred in 8.2% (74/903) of cases. The first framing coil was completely conformed to the lesion morphology in 83.3% (724/869) of cases. The immediate post-procedure RROC Class I or II was reported in 79.7% (717/900) of all aneurysm cases and at 1-year was 90% (641/712). The overall 1-year retreatment rate was 7.1% (52/731) and the recanalization rate was 12.4%. RROC I or II at 1-year was achieved in 84.3% (161/191) of ruptured aneurysms and in 92.1% (480/521) of unruptured aneurysms. The retreatment rate for ruptured aneurysms (*N* = 288) at 1-year was 16.5% (33/200) and for unruptured aneurysms was 3.6% (19/531). The retreatment rate for wide-neck aneurysm (WNA, *N* = 554) at 1-year was 7.4% (34/458) compared to 6.4% (16/251) for non-WNA. Supplementary Table 1 shows the outcomes for RROC at 1-year by immediate post-procedure RROC.

#### Safety Outcomes

Procedural device-related SAEs occurred in 2.9% (26/905) of patients and one patient died within 24 h of the procedure (described above in “All Patient Results”). The mortality rate at 1-year was 5.4% (49/905); 11.8% for patients with ruptured aneurysms and 2.4% for patients with unruptured aneurysms. At 1-year, there were 84.4% (429/508) of patients with mRS 0-2; 65.3% for ruptured aneurysms and 92.2% in unruptured aneurysms. The procedural device-related SAE rate for patients treated for ruptured aneurysms was 3.1% (9/288) and for unruptured aneurysms was 2.8% (17/617). In patients with WNA, the procedural device-related SAEs rate was 2.5% (n/*N*) and this rate for non-WNA was 3.8% (12/318).

#### Predictive Analysis

Predictors of 1-year for RROC III or retreatment (incomplete occlusion) were rupture status (OR 3.61, 95% CI 2.27–5.74, *P* < 0.0001), balloon-assisted coiling (OR 1.72, 95% CI 1.04–2.87, *P* = 0.0354), aneurysm size (OR 2.23, 95% CI 1.24–4.00, *P* = 0.0071), and incomplete occlusion immediate post-procedure (OR 2.01, 95% CI 1.19–3.37, *P* = 0.0086). Predictor of aneurysm retreatment at 1-year was rupture status (OR 5.32, 95% CI 2.95–9.61, *P* < 0.0001).

### Non-aneurysm Cohort Outcomes

Of the 90 patients treated for non-aneurysms; 45.6% (41/90) were fistulas, 12.2% (11/90) were arteriovenous malformations, and 42.2% (38/90) were vessel sacrifice or pseudoaneurysm. In non-aneurysm treated patients, UAC was used in 60.0% (54/90), liquid embolics in 22.2% (20/90), and 10.0% (9/90) particulate/plug embolics. 79.2% (42/53) of all patients had the first coil conformed to the lesion. The median time from first coil to last coil deployment was 20.5 min (IQR 3.0, 53.0) and the median fluoroscopy time was 41.0 min (IQR 25.0, 73.0). Retreatment at 1-year was 1.9% (1/53) and the recanalization rate was 6.1% (3/49). At 1-year, 90.9% (20/22) of fistula treated patients, 54.5% (12/22) of patients treated for other lesions, and 40% (2/5) of patients treated for malformation had mRS 0-2. There were no procedural device-related SAEs (0.0%) and the mortality rate within 24 h of the procedure was 1.1% (1/90). The mortality rate at 1-year was 12.2% (11/90).

## Discussion

The results of the SMART Registry provide clinical evidence of the treatment with SMART, PC400, and POD in intracranial aneurysms and other neurovascular abnormalities. Patients in this registry had low retreatment rates through 1 year. The procedural device-related SAE rate was low. A high rate of patients achieved adequate occlusion post-procedure and at 1-year, and our rates were consistent or better than rates reported in other SMART COIL series (5–8, 11). Mortality within 24 h of the procedure occurred in two patients due to progression of baseline SAH from a ruptured aneurysm. The SMART Registry results confirm the safety and durability of the SMART System for treatment of intracranial aneurysms and other neurovascular abnormalities.

The SMART Registry outcomes are comparable to contemporary studies of other coils. The TARGET Registry was a prospective, single-arm registry that compared outcomes for patients with saccular (ruptured and unruptured) intracranial aneurysms treated exclusively with TARGET-360° (Stryker Corp., Fremont, California) complex shape coils (designated for framing, filling, and finishing) vs. patients treated with both complex shape and helical coils (12). There were no significant differences between the groups for complete or near complete occlusion and in retreatment rates at 6 months, or for any other safety or efficacy outcomes. In the combined group analysis, median packing density was 28.8%, 6-month RROC I or II was 90.4%, and recanalization was 15.2%. In comparison to the SMART Registry, the mean packing density was 32.3%; and at 1-year, RROC I or II was 90% and recanalization was 12.9%. Despite the SMART Registry having longer follow-up than the TARGET Registry (12 ± 6 months), the SMART Registry occlusion rate was comparable and the recanalization rate was lower than in TARGET registry. In addition, TARGET excluded fusiform and dissecting aneurysms as well as patients with severe aneurysm ruptures (Hunt and Hess > 3), while in the SMART Registry 20.5% (58/283) of patients had severe aneurysm ruptures.

In the prospective randomized GREAT Trial, intracranial aneurysms ranging 4–12 mm diameter were treated with either second generation hydrogel coils or platinum coils (13). There were no significant differences in procedural outcomes between the two coil types. The angiographic follow-up at 18 months showed that the platinum coil arm had adequate occlusion (per core lab RROC I + II) in 73% of patients and retreatment rate of 6%. The hydrocoil arm had higher adequate occlusion (80%) and a lower retreatment rate (3.0%) than the platinum coil arm. In comparison, the SMART Registry rate of adequate occlusion at 12 months was higher (90.0%) than both treatment arms in GREAT and retreatment rates for the SMART Registry and GREAT were similar. In a large systematic review including 8,161 aneurysms treated with standard and modified (hydrocoils and coated) coils, the retreatment rate was 10.3% (95% CI 9.5%, 11.0%) (14).

The SMART Registry provides evidence of safety of treatment in aneurysms and other neurovascular lesions. Mortality within 24 h of the procedure in the overall population was low (0.2%) and none were procedural or device-related. In the TARGET real-world randomized trial that evaluated outcomes after coiling aneurysms, the periprocedural mortality rate was 0.7% (12). In the GREAT trial for aneurysms, the 14-day mortality was 2.1% in the bare platinum coil arm (13). In the SMART Registry, there were no device complications associated with an AE and safety was demonstrated across multiple complex lesion types. In the non-aneurysm subgroup of the SMART Registry, which included fistulas, arteriovenous malformations, pseudoaneurysms, and vessel sacrifices, there were no procedural device-related SAEs, adequate occlusion immediate post-procedure was high (96.6%), and retreatment at 1-year was low (1.9%). These results are consistent with previous POD studies and also alternative treatments (15–17).

The SMART Registry included several complex lesion subgroups, such as a large number of patients treated for wide-neck aneurysms (WNA), ruptured aneurysms, and patients with non-saccular aneurysms (e.g., fusiform and dissecting). The patients treated for WNA had low procedural device-related safety events (2.5%) and low retreatment rates (7.4%) through 1-year. These outcomes are consistent with other reports of coiled WNA (18–23). Patients with ruptured aneurysms in our study achieved high occlusion rates at 1-year (RROC I + II 84.3%) and as expected, these patients had higher rates of recanalization and retreatment compared to those patients treated for unruptured aneurysms. These findings are consistent with previous reports in real-world registries treating ruptured aneurysms with endovascular coiling (24–26). The SMART ruptured aneurysm subgroup achieved occlusion comparable to other studies evaluating ruptured aneurysms treated with bare metal coils (24). Another study of ruptured aneurysms treated with coiling reported a periprocedural safety complication rate as high as 12.7% compared to 3.1% in the SMART Ruptured population (27). Therefore, SMART Coil appears to maintain safety and performance across multiple complex lesion types. Further investigation into complex subgroups from this study will be presented in subsequent reports from the SMART Registry.

The unique design of the SMART System features progressive coil softness and polymer technology that are not present in other commercially available coils. The coil tip is a softened loop and is designed to help reduce kickback and vessel perforations. The progressive coil softness potentially can lead to less compartmentalization and better packing density (28). Rigid coils are typically effective for framing, while softer coils are more effective for filling and finishing. The SMART COIL design decreases compartmentalization with stable framing of the aneurysm wall and supports durable occlusion through 1 year. In the SMART Registry, compartmentalization rate was low (2.0%) and demonstrated the ability to treat a variety of complex lesion types and treat distal lesion locations safely, such as the anterior, mid, and posterior communicating arteries. Previous studies have suggested a correlation between coil packing density and recanalization (29). Increased packing density promotes accelerated healing and decrease recurrence and retreatment (30). In other neurovascular embolization coil studies, packing density has been reported between 27.4 and 37.9% (7, 31–34). The mean packing density was 32.3% in the SMART Registry and this may have contributed to low rates of recanalization.

In this study population, we found that predictors at 1-year for retreatment was ruptured status. RROC III immediate post-procedure, aneurysm size, balloon-assisted coiling, and ruptured aneurysm were predictors for RROC III or retreatment (incomplete occlusion) at 1-year. The predictors for occlusion in the TARGET Registry were aneurysm location, size, and immediate occlusion grade (12).

The SMART Registry is one of the largest real-world prospective embolization coil studies completed to date and included a large sample size of complex lesions. The study included ruptured and unruptured aneurysms ranging from small to giant sizes, wide-neck and complex shaped aneurysms; and other malformations, including arterial fistula, malformations, and vessel embolization for treatment of tumors or hemorrhages. Heterogenicity of the patient and lesion population allows future insight into multiple complex subgroups and provides good reflection of current treatment techniques and long-term outcomes using contemporary coils. A key limitation of the study is the lack of a comparator arm. Another limitation was that this study did not have a centralized angiographic core lab performing independent occlusion assessments.

## Conclusion

This study provides clinical evidence of the SMART System in real-world clinical settings. The findings demonstrated that the SMART System is safe and efficacious and substantiate the durability of the coils at 1-year. Additional analysis of subgroups will be beneficial.

## Data Availability Statement

The original contributions presented in the study are included in the article/Supplementary Material, further inquiries can be directed to the corresponding author/s.

## Ethics Statement

The studies involving human participants were reviewed and approved by all investigational study sites received approval and oversite from their ethics committee. The patients/participants provided their written informed consent to participate in this study.

## Author Contributions

All authors were responsible for conducting research, data collection, analysis, interpretation, review of the article, final draft approval, and primary investigators at their institutions and were responsible for protocol execution within local and international regulatory requirements. AS was the Principal Investigator and lead author.

## Conflict of Interest

This study was funded by Penumbra Inc. (Alameda USA). Both the sponsor and authors were involved in the design and conduct of the study; collection, management, analysis, and interpretation of the data; preparation, review, and approval of the manuscript; and decision to submit the manuscript for publication. The reviewer AC declared a past co-authorship with one of the authors OZ to the handling editor.
