# Supplementary material for: The SMART Registry: Long-Term Results on the Utility of the Penumbra SMART COIL System for Treatment of Intracranial Aneurysms and Other Malformations
Source: Front Neurol. 2021 Apr 13;12:637551. doi: 10.3389/fneur.2021.637551 (PMC8076606; doi:10.3389/fneur.2021.637551)
Supplement: Supplementary file 1 [file Table_1.docx]

**Supplementary Materials**

**Supplementary Table 1: Study Aneurysm Outcomes Based on RROC^*^**

| **Immediate Post-Procedure** | **RROC I at 1-Year** | **RROC II at 1-Year** | **RROC III at 1-Year** |
| --- | --- | --- | --- |
| **RROC I**  39.4% (279/708) | 78.1% (218/279) | 16.5%  (46/279) | 5.4%  (15/279) |
| **RROC II**  39.1% (277/708) | 54.9%  (152/277) | 34.3% (95/277) | 10.8% (30/277) |
| **RROC III**  21.5% (152/708) | 57.2%  (87/152) | 25.7%  (39/152) | 17.1%  (26/152) |

***Occlusion was determined by the Treating Physician**

**Supplementary Table 2: List of SMART Investigators**

| **Investigators** | **Hospital** |
| --- | --- |
| PI Andrew Ducruet (PI)  Bruno Flores (Sub-I)  Dale Ding (Sub-I)  Felipe Albuquerque (Sub-I)  Alfred Pokmeng See (Sub-I)  Rami Almefty (Sub-I) | St. Joseph's Hospital and Medical Center |
| Frank Hellinger (PI)  Ravi Gandhi (Sub-I) | Florida Hospital Orlando |
| Richard Bellon (PI) Donald Frei (Sub-I)  Ian Kaminsky (Sub-I)  Benjamin Atchie (Sub-I)  David Loy (Sub-I) | Swedish Medical Center |
| PI Paul Jacobson (PI)  Daniel Reed Hoss (Sub-I) | Loma Linda University Health |
| Min Park (PI)  Avery Evans (Sub-I)  Robert Hixson (Sub-I)  Jean-Martin Gingras (Sub-I)  Thomas Buell (Sub-I)  Yashar Kalani (Sub-I) | University of Virginia |
| Sohyun Boo (PI)  Ann Noelle Lucke-Wold (Sub-I)  Ansaari Rai (Sub-I) | West Virginia University Hospital |
| David Fiorella (PI)  Eugene Gu (Sub-I) | Stony Brook University |
| Yin Hu (PI)  Jonathon Pace (Sub-I)  Abhishek Ray (Sub-I)  Mickey Smith (Sub-I)  Jeffery Nelson (Sub-I) | University Hospitals Cleveland Medical Center |
| Brijesh Mehta (PI)  Hoang Duong (Sub-I)  Gina DiMartini (Sub-I)  Andrey Lima (Sub-I) | Memorial Hospital West |
| Tarun Bhalla (PI) | Strong Memorial Hospital (Univ. of Rochester) |
| Michael Korona (PI)  James Martin (Sub-I)  Paul Blom (Sub-I) | St. Mary’s Medical Center |
| Scott Simon (PI) | The Milton S. Hershey Medical Center |
| Dan Victor Giurgiutiu (PI) | Valley Health/Winchester Medical Center |
| Alejandro Spiotta (PI)  Jonathan Lena (Sub-I)  Aquilla Turk (Sub-I)  Raymond Turner (Sub-I)  Jan Vargas (Sub-I)  Mohammad Imran Chaudry (Sub-I) | Medical University of South Carolina (MUSC) |
| Avi Setton (PI) | North Shore University Hospital |
| Andrew Ku (PI)  Eddie Sui Ki Kwan (Sub-I)  Richard Williamson (Sub-I) | Allegheny General Hospital |
| Keith Woodward (PI)  Harry Hixson (Sub-I) | Fort Sanders Regional Medical Center |
| Cole Graham (PI)  Roham Moftakhar (Sub-I) | Palmetto Health Richland Hospital |
| Alois Zauner (PI)  Robert Taylor (Sub-I) | Santa Barbara Cottage Hospital |
| Theodore Larson (PI) | St. Anthony Hospital |
| Michael Hurley (PI)  Ali Shaibani (Sub-I)  Matthew Potts (Sub-I)  Sameer Ansari (Sub-I)  Babak Jahromi (Sub-I) | Northwestern University |
| Richard Klucznik (PI)  Orlando Diaz (Sub-I)  Yi Zhang (Sub-I) | Houston Methodist Hospital |
| Zinovy Katz, MD (PI) | Bayfront Health St. Petersburg |
| Rabih Tawk, MD (PI)  David Miller, MD (Sub-I)  Benjamin Brown, MD (Sub-I) | Mayo Clinic Jacksonville |
| Shakeel Chowdhry, MD (PI) | Evanston Hospital |
| Alan Reeves, MD (PI)  Koji Ebersole (Sub-I)  Michael Abraham (Sub-I)  Ernest John Madarang (Sub-I) | University of Kansas Medical Center |
| Reade DeLeacy (PI)  J Mocco (Sub-I)  Johanna Fifi (Sub-I) | Mount Sinai Hospital |
| Bharathi Jagadeesan (PI) | University of Minnesota Medical Center – Fairview |
| Kenneth Snyder (PI)  Adnan Siddiqui (Sub-I)  Gursant Atwal (Sub-I)  Jason Davies (Sub-I)  Jeffrey Beecher (Sub-I)  Kunal Vakharia (Sub-I)  Vernard Fennell (Sub-I)  Elad Levy (Sub-I) | Buffalo General Hospital |
| Dr. Zeguang Ren (PI)  Dr. Maxim Mokin (Sub-I) | Tampa General Hospital |
| Dr. Richard Bellon (PI)  Dr. Donald Frei (Sub-I)  Dr. David Loy (Sub-I)  Dr. Benjamin Atchie (Sub-I) | Lutheran Medical Center |
| Dr. Thomas Wolfe (PI) | Aurora St. Luke's Medical Center |
| Dr. Vicken Garabedian (PI)  Dr. Matthew Ponds (Sub-I) | St. Alphonsus Regional Medical Center |
| Harris Hawk (PI)  Blaise Baxter (Sub-I)  Steven Quarfordt (Sub-I)  Justin Calvert (Sub-I) | Erlanger Health System |
| Robert (Bobby) Starke (PI)  Dileep Yavagal (Sub-I)  Eric Peterson (Sub-I) | Jackson Health System |
| Robert James (PI) | University of Louisville Hospital |
| Paul Saphier (PI)  Beth Karasin (Sub-I)  Lauren Eskuchen (Sub-I)  Ronald Benitez (Sub-I) | Overlook Medical Center |
| David Case (PI)  Joshua Seinfeld (Sub-I)  Sheila Jo Kubes (Sub-I)  Jason Rich (Sub-I)  Christopher Roark (Sub-I) | University of Colorado Hospital |
| Darryn Shaff (PI) | Lehigh Valley Hospital |
| Kyle Fargen (PI)  Stacey Quintero Wolfe (Sub-I)  Jasmeet Singh (Sub-I) | Wake Forest University Health Sciences |
| Brian Kott (PI) | MultiCare Tacoma General Hospital |
| Stavropoula Tjoumakaris (PI) Pascal Jabbour (Sub-I)  Robert Rosenwasser (Sub-I)  Nabeel Herial (Sub-I)  Michael Reid Gooch (Sub-I) | Thomas Jefferson University |
| Athos Patsalides (PI)  Jared Knopman (Sub-I)  Srikanth Boddu (Sub-I) | Weill Cornell Medical College |
| Christopher Nickele (PI)  Adam Arthur (Sub-I)  Daniel Hoit (Sub-I)  Lucas Elijovich (Sub-I)  Violiza Inoa (Sub-I) | Methodist University Hospital |
| Ali Malek (PI) | St. Mary's Medical Center (Tenet) |
| Patrick Brown (PI)  Andrew Ferrell (Sub-I)  Peter Kvamme (Sub-I) | University of Tennessee Medical Center |
| Lucian Maidan (PI)  George Luh (Sub-I) | Mercy San Juan Medical Center |
| Peter Sunenshine (PI)  Karam Moon (Sub-I) | Banner University Medical Center – Phoenix |
| Bharathi Jagadeesan (PI) | Hennepin County Medical Center |
| Scott Geraghty (PI)  Hamad Farhat (Sub-I)  Thomas Grobelny (Sub-I) | Advocate Christ Medical Center |
| Albert Yoo (PI)  Alexander Venizelos (Sub-I)  Aashish Anand (Sub-I)  Benny Kim (Sub-I)  Jazba Soomro (Sub-I)  Naregnia Pierre-Louis (Sub-I)  Norman Ajiboye (Sub-I)  Parita Bhuva (Sub-I)  Paul Hansen (Sub-I)  Ryan Gianatasio (Sub-I) | Medical City of Plano |
| Lissa Peeling (PI)  Gary Hunter (Sub-I)  Michael Kelly (Sub-I) | Royal University Hospital |
| Lucas Elijovich (PI)  Adam Arthur (Sub-I)  Christopher Nickele (Sub-I)  Daniel Hoit (Sub-I)  Violiza Inoa (Sub-I) | Baptist Memorial Hospital |
| Thinesh Sivapatham (PI)  Barbara Albani (Sub-I)  Gregg Zoarski (Sub-I)  Sudhakar Satti (Sub-I) | Christiana Care Health Services |
| Amir Khan (PI)  Armen Choulakian (Sub-I) | UCSF – Fresno |
| Ashish Nanda (PI)  Sushant Kale (PI)  Amer Alshekhlee (Sub-I) | SSM St. Clare Healthcare |
| Bradley Bohnstedt (PI)  Ankur Garg (Sub-I)  Sudeepta Dandapat (Sub-I)  Vijay Pandav (Sub-I) | University of Oklahoma  Health Sciences Center |
| Abhineet Chowdhary (PI) | Overlake Hospital Medical Center |
| John Chaloupka (PI) | Mount Sinai Medical Center |
| Eric Sauvageau (PI)  Amin Aghaebrahim (Sub-I)  Ricardo Hanel (Sub-I) | Baptist Medical Center |
| Mouhammed Kabbani (PI) | Gundersen Clinic |
| Yahia Lodi (PI)  Varun Reddy (Sub-I)  Qingliang Wang (Sub-I) | UHS Wilson Medical Center |
| Clemens Schirmer (PI)  Shamsher Dalal (Sub-I)  Oded Goren (Sub-I)  Christoph Griessenauer (Sub-I) | Geisinger Health System |
| Augusto Elias (initial PI)  Robert Burke (Sub-I)  Casey Muehle (Sub-I, then PI) | Memorial Medical Center |
| Ameer Hassan (PI)  Wondwossen Tekle (Sub-I) | Valley Baptist Medical Center |
| Augusto Elias (PI)  Ravi Shastri (Sub-I) | Metro Health – University of Michigan Health |
| Osama Zaidat (PI)  Eugene Lin (Sub-I)  Bader Alenzi (Sub-I)  Mohammad Ezzeldin (Sub-I) | Mercy Health St. Vincent Medical Center |
| Yafell Serulle (PI) | Westside Regional Medical Center |
| Rishi Gupta (PI)  Ahmad Khaldi (Sub-I) | WellStar Kennestone Hospital |
| Travis Dumont (PI)  Leonardo Brasiliense (Sub-I) | Banner University Medical Center – Tucson |
| Ritesh Kaushal (PI) | Palmetto General Hospital (Tenet) |
| Mohamed Teleb (PI) | Banner Desert Medical Center |
| Akram Shhadeh (PI)  Vikas Gupta (Sub-I) | Lawnwood Regional Medical Center |
| Joshua Bentley (PI) | Southeast Alabama Medical Center |
| Varun Reddy (PI)  Qingliang Wang (Sub-I)  Yahia Lodi (Sub-I) | Faxton - St. Luke's Healthcare |
